# 2217. Evaluation of Methicillin-Resistant *Staphylococcus aureus* (MRSA) Nasal Swab Screening in Patients with Cancer at a Large Comprehensive Cancer Center

**DOI:** 10.1093/ofid/ofad500.1839

**Published:** 2023-11-27

**Authors:** Mark Herrington, Nancy N Vuong, Chun Feng, Hyunsoo Hwang

**Affiliations:** The University of Texas MD Anderson Cancer Center, Houston, Texas; The University of Texas MD Anderson Cancer Center, Houston, Texas; The University of Texas MD Anderson Cancer Center, Houston, Texas; The University of Texas MD Anderson Cancer Center, Houston, Texas

## Abstract

**Background:**

Methicillin-resistant *Staphylococcus aureus* (MRSA) nasal screening has a high negative predictive value (NPV) in immunocompetent patients with infections and is widely utilized as a tool for antibiotic discontinuation. The data applying nasal swab screening in patients with cancer is limited, particularly for those that are immunocompromised and in the setting of neutropenic fever. The aim of this study is to determine the predictive values of MRSA swabs screenings in patients living with cancer.

**Methods:**

This is a retrospective cohort observational study of adult patients admitted to The University of Texas MD Anderson Cancer Center between January 2019 through October 2022. Data from patients with documented MRSA nasal swab screenings and clinical cultures taken within 7 days was collected. The first documented MRSA swab screening and culture results from unique patients were included for analysis to calculate sensitivity, specificity, positive predictive value, and NPV.

**Results:**

A total of 6,475 patients with MRSA swab screenings had 13,129 clinical cultures taken from different anatomical sites. Of the patients included, 57% had a solid tumor and 37% had a hematological malignancy, with 82% of patients receiving an anti-MRSA antibiotic agent within 90 days of the MRSA nasal swab. There were 167 documented positive MRSA cultures, most commonly from a wound (41.3%) or respiratory source (24%). Overall sensitivity and specificity for all culture sites were 50.9% and 98.4%, respectively, with an overall NPV of 99.4%. The NPV was 99.8% for bloodstream infections, 98.5% for respiratory infections, 92.6% for wound infections, and greater than 99% for other culture sites.

**Conclusion:**

The specificity and negative predictive value of MRSA swab screenings in patients with cancer was high overall and consistent with the literature in immunocompetent patients. These results can help guide in the discontinuation of empiric antibiotics in patients living with cancer.

**Disclosures:**

**All Authors**: No reported disclosures

